# The relationship of lamins with epigenetic factors during aging

**DOI:** 10.18699/VJGB-22-06

**Published:** 2022-02

**Authors:** R.N. Mustafin, E.K. Khusnutdinova

**Affiliations:** Bashkir State Medical University, Ufa, Russia; Institute of Biochemistry and Genetics – Subdivision of the Ufa Federal Research Centre of the Russian Academy of Sciences, Ufa, Russia

**Keywords:** lamins, microRNAs, transposons, epigenetic factors, ламины, микроРНК, транспозоны, эпигенетические факторы

## Abstract

The key factor of genome instability during aging is transposon dysregulation. This may be due to senile changes in the expression of lamins, which epigenetically modulate transposons. Lamins directly physically interact with transposons. Epigenetic regulators such as SIRT7, BAF, and microRNA can also serve as intermediaries for their interactions. There is also an inverse regulation, since transposons are sources of miRNAs that affect lamins. We suggest that lamins can be attributed to epigenetic factors, since they are part of the NURD, interact with histone deacetylases and regulate gene expression without changing the nucleotide sequences. The role of lamins in the etiopathogenesis of premature aging syndromes may be associated with interactions with transposons. In various human cells, LINE1 is present in the heterochromatin domains of the genome associated with lamins, while SIRT7 facilitates the interaction of this retroelement with lamins. Both retroelements and the nuclear lamina play an important role in the antiviral response of organisms. This may be due to the role of lamins in protection from both viruses and transposons, since viruses and transposons are evolutionarily related. Transposable elements and lamins are secondary messengers of environmental stressors that can serve as triggers for aging and carcinogenesis. Transposons play a role in the development of cancer, while the microRNAs derived from them, participating in the etiopathogenesis of tumors, are important in human aging. Lamins have similar properties, since lamins are dysregulated in cancer, and microRNAs affecting them are involved in carcinogenesis. Changes in the expression of specif ic microRNAs were also revealed in laminopathies. Identif ication of the epigenetic mechanisms of interaction of lamins with transposons during aging can become the basis for the development of methods of life extension and targeted therapy of age-associated cancer

## Abstract (ru)

Дисбаланс активации транспозонов – один из важнейших факторов нестабильности генома при старении. Причинами этого явления могут быть ассоциированные с возрастом изменения экспрессии ламинов, которые влияют на эпигенетическую регуляцию мобильных генетических элементов. Взаимосвязь ламинов и транспозонов может быть обусловлена специфическим физическим контактом между молекулами, а также опосредована эпигенетическими регуляторами, такими как SIRT7, BAF и микроРНК. Характерна взаиморегуляция ламинов с мобильными элементами, которые являются источниками микроРНК, влияющими на ламины. Ламины входят в состав NURD (nucleosome remoldeling deacetylase complex), взаимодействуют с гистоновыми деацетилазами и регулируют экспрессию генов без изменения структуры нуклеотидных последовательностей. Роль ядерной ламины в этиопатогенезе синдромов преждевременного старения может быть обусловлена взаимодействием с транспозонами, так как истощение ламинов приводит к активации мобильных генетических элементов. В различных клетках человека LINE1 представлены в связанных с ламинами гетерохроматиновых доменах генома, при этом SIRT7 способствует взаимодействию данного ретроэлемента с ядерной ламиной. В противовирусном ответе организмов важную роль играют как ретроэлементы, так и ядерная ламина. Это согласуется с ролью ламинов в защите как от вирусов, так и от транспозонов, которые характеризуются филогенетическим родством. Мобильные генетические элементы и ламины – вторичные мессенджеры средовых стрессорных воздействий, которые могут служить пусковыми факторами для старения и канцерогенеза. Транспозоны играют роль в развитии злокачественных новообразований, при этом происходящие от них микроРНК, участвующие в этиопатогенезе опухолей, имеют значение в старении человека. Сходные свойства типичны для ламинов, поскольку при злокачественных новообразованиях выявлена дисрегуляция ламинов, а влияющие на них микроРНК участвуют в канцерогенезе. Изменение экспрессии специфических микроРНК отмечено также при ламинопатиях. Определение точных эпигенетических механизмов взаимодействия ламинов с мобильными генетическими элементами при старении может стать основой для разработки методов продления жизни и таргетной терапии ассоциированных с возрастом злокачественных новообразований.

## Introduction

Nuclear lamina (NL) is a protein network connected to the inner side of the cell’s nuclear envelope. Nuclear lamina performs structural, signaling and regulatory functions. The study of the evolution of NL components made it possible to discover a wide variety of domains and sequence architectures that go beyond the classical alpha-helices (Kollmar, 2015). Nuclear lamina is composed of lamins and associated proteins (Lemaitre, Bickmore, 2015). The main components of NL are lamins (Cibulka et al., 2012). Their genes were present at the earliest stages of eukaryotic evolution. Lamins have been identified in multicellular organisms, amoebas, and primitive opistocontes such as Ichthyosporea and Choano-f lagellates (Kollmar, 2015). Lamins are required not only to maintain the shape of the nucleus, but also to control replication and transcription. Lamins are proteins of the family of intermediate filaments (class V) with a specific structure. In mammalian cells, 4 types of lamins are expressed: A and C (splicing isoforms of one gene), B1 and B2 (Cibulka et al., 2012). Minor isoforms Аδ10 and С2 are also known. The protein precursor of lamin A is prelamin A. Its C-terminal region is post-translationally modified: farnesylated, carboxymethylated. Proteolysis of the CaaX motif of pre-lamin A also occurs using the metalloprotease ZMPSTE24 (encodes a metalloproteinase that metabolizes lamin A) (Wang et al., 2016). Lamins A, С, Аδ10 and С2 are encoded by LMNA gene (Turgay et al., 2017).

Proteins that interact with lamins and are closely associated with them functionally include LBR (lamin B receptor), BAF (barrier to autointegration factor), SUN1, SUN2, nesprin. They are involved in the structural organization of the nucleus and in the regulation of nuclear processes. LBR and endoprotease prelamin A have enzymatic properties (Meinke et al., 2014).

At the periphery of the nucleus, genomic DNA binds to lamins A and B, forming heterochromatic domains. Lamin A also binds to chromatin inside the nucleus – the nucleoplasmic environment is represented mainly by euchromatin, which indicates the role of lamin A in the regulation of gene expression of the entire genome (Briand et al., 2018). Lamin B interacts with genomic domains that are relatively gene-poor and transcriptionally inactive (Guelen et al., 2008). It is assumed that in the last common predecessor of all eukaryotes (LECA – last eukaryotic common ancestor), the configurations of the nuclear envelope and the associated NL played an important role in determining the activity of the nucleus.

In the subsequent evolution, there were changes in the mechanisms of regulation of gene expression of lamins in different taxa. The protein diversity of lamins in plants and trypanosomes is taxonomically limited, while in multicellular animals it is manifested in a wider range. The phylogenetic tree of lamina genes is characterized by vertical evolution. For example, two lamins in protists from strongly diverging taxa have targets in mammalian cell nuclei and polymerize into filamentous structures, which indicates the functional preservation of distant homologues of lamins. In certain groups of eukaryotes, a pronounced evolutionary plasticity of the structures of the NL was determined by the mechanisms of chromatin binding and epigenetic control due to a high level of divergence of lamins’ homologues (Korney, Field, 2016).

Lamin changes occur with aging and in age-associated diseases such as malignant neoplasms. For example, in lung cancer, significant reductions in lamin B1 levels are found (Garvalov et al., 2019). Lamin A regulates the activity of mTOR (mechanistic target of rapamycin), the low activity of which contributes to an increase in life expectancy. Inhibition of mTOR (for example, via rapamycin) leads to the degradation of defective molecules and organelles that accumulate in cells during aging, since low autophagy activity is a characteristic feature of aging-related diseases (Cenni et al., 2020).

Lamins can be attributed to epigenetic factors, since they ensure the inheritance of the functional status of the gene. For example, the attachment of lamin B1 to specific genome loci leads to transcription suppression of genes located in them. Most differentially expressed genes are activated due to the loss of lamin B1. These include the genes of the RET oncogene and its corepressor GFRα1. Therefore, when lamin B1 is depleted with aging, the risk of tumors increases. Another mechanism of RET activation is H3K27me3 histone methylation (Garvalov et al., 2019).

Epigenetic factors include chromatin remodeling, modifications of histones and DNA nucleotides, and the influence of non-coding RNAs (ncRNAs). During cellular senescence in mammals, H3K56ac and H4K16ac levels decrease, which contributes to altered gene expression, genomic instability, and telomere damage. Aging is accompanied by a global decrease in DNA methylation levels, coinciding with agerelated loss of heterochromatin. As a result, the regulation of gene expression and activation of transposable elements (ТЕs) is disrupted. At the same time, the participation of lamins in epigenetic regulation associated with aging occurs through direct interaction with chromatin in regions of specific DNA sequences called lamin-associated domains (LADs).

Lamins also promote epigenetic changes during aging through functional interactions with sirtuins. For example, lamin A enhances the deacetylase activity of SIRT1, stimulates the functioning of SIRT6 in DNA repair, and recruits histone deacetylase 2 (HDAC2). Lamins A/C are also direct participants in epigenetic regulation, since they serve as components of the NURD (nucleosome remodeling deacetylase complex). This complex also includes HDAC1, RBBP4, RBBP7 (Cenni et al., 2020). Lamins A/C interact with histone deacetylase 2 (HDAC2) and with PCAF acetyltransferase (p300-CBP associated factor) (Santi et al., 2020). Lamin B1 recruits the PRC2 (Polycomb repressive complex 2) complex, through which the H3K27me3 landscape changes with repression of specific genes involved in signaling and cell migration (Jia et al., 2019).

## The role of lamins in premature aging syndromes

The development of progeria in patients with germinal mutations in the genes of lamins proves the role of lamins in regulation of life expectancy. Progeroid laminopathies (PLs) are characterized by premature aging and death from complications of atherosclerosis such as myocardial infarction, stroke, or heart failure. PLs are usually not inherited because patients do not survive to puberty.

An important genetic feature of progeria is telomere shortening with each replication cycle (Ahmed et al., 2018). The most famous of these diseases is Hutchinson–Guildford progeria (HGP), which is characterized by significant telomere shortening. The disease is diagnosed from two years of age, when noticeable symptoms of premature aging are determined. Life expectancy in HGP patients is 10–20 years. In 90 % of HGP patients, mutations in the LMNA gene are determined (Ahmed et al., 2018). The frequency of occurrence of HGP is 1 in 8 million newborns (Burla et al., 2018). The most common mutation in LMNA gene is C1824T, which leads to accumulation of progerin (the dominant negative form of lamin A).

Interestingly, progerin also accumulates in cells during physiological aging as one of the rare splicing forms of lamin A transcripts. However, in comparison with physiological aging, more severe epigenetic changes occur in HGP, such as covalent modifications of histones with a tendency to loss of separation into hetero- and euchromatin. These epigenetic changes lead to changes in the spatial compartmentalization and conformation of chromatin in the nucleus. These processes involve microRNAs, such as miR-9, which can be used as a target for brain protection in HGP patients (Arancio et al., 2014). MiR-9 interacts with the 3′-untranslated region (UTR) of lamin A mRNA without affecting lamin C. 3′-UTR region of prelamin A contains an additional binding site for miR-9. In experiments on mice and human HeLa cell cultures, it was proved that miR-9 expression significantly reduces the levels of lamin A (Jung et al., 2012).

As with normal aging, abnormal accumulation of progerin suppresses the interactions of lamin A with SIRT1, HDAC2, and SIRT6. In addition, in patients with HGP, the regulation of the heterochromatin protein HP1 is impaired, the levels of H3K9me3 decrease, and the NURD function is suppressed (of which lamin A/C is a component). At the same time, the relationship of lamin A/C with HDAC2 causes the activation of the CDKN1A gene, which is the most important determinant of cellular senescence (Cenni et al., 2020).

Mutations in LMNA gene also cause atypical Werner’s syndrome (AWS), mandibuloacral dysplasia type A (MADA), atypical progeria syndrome (APS) (Burla et al., 2018). They differ in disease-specific mutations in LMNA gene and in clinical manifestations. Dunnigan-type familial partial lipodystrophy is caused by an R482W missense mutation in lamin A, leading to a pathological change in its effect on the periphery and inside the nucleus on a three-dimensional rearrangement of chromatin (Briand et al., 2018). MADA can be caused by mutations in the LMNA or ZMPSTE24 genes. It is a rare autosomal recessive disorder characterized by bone abnormalities with localized osteolysis and generalized osteoporosis, skin pigmentation, lipodystrophy, and accelerated aging. These mutations cause accumulation of prelamin A, which leads to disruption of chromatin dynamics. Similar changes are found during physiological aging (Cenni et al., 2018).

Progeroid laminopathies are also caused by mutations in genes that interact with lamins. For example, Nestor– Guillermo progeria (NGPS) is caused by mutations in the BAF gene (Loi et al., 2016). Premature aging syndromes can be caused not only by mutations in the genes of lamins and proteins interacting with them, but also by mutations in the genes of DNA repair and maintenance enzymes. For example, mutations in helicase genes cause Rothmund– Thomson syndrome (RECQL4 gene), Bloom syndrome (BLM gene), Werner syndrome (WRN gene). Mutations in DNA repair genes cause Dyskeratosis congentia (DKC1, TERC, TERT genes) and Cockayne syndrome (ERCC8 or ERCC6 genes). In contrast to these premature aging syndromes, PLs are characterized by an early onset, more severe manifestations of aging and a lack of predisposition to cancer (Burla et al., 2018). All PLs are characterized by generalized osteoporosis and osteolysis, crowding of teeth with malocclusion. Progeroid laminopathies with a specific lesion of the musculoskeletal system include heart-hand syndrome of Slovenian (HHS-S) (Gargiuli et al., 2018). Mutations in the ZMPSTE24 gene are causes of the development of restrictive dermopathy (RD) and mandibuloacral dysplasia type B (MADB) (Burla et al., 2018).

In ataxia-telangiectasia patients, accumulation of lamin B1 causes changes in the shape of the nucleus and cell aging. Oxidative stress in this syndrome increases lamin B1 levels by stimulating mitogen-activated protein kinase p38 (p38 MAPK). In cell experiments using the MAPK activator anisomycin and the MAPK inhibitor SB203580 to determine the effect of p38 MAPK on lamin B1 levels, p38 MAPK activation was shown to significantly increase lamin B1 levels. At the same time, inhibition of p38 MAPK decreases lamin B1 levels. Using the PLA method (proximity ligation assay), it was proved that p38 MAPK interacts with lamin B1, causing its phosphorylation (Barascu et al., 2012). These changes also occur during natural aging. Also, PLs are characterized by decrease in perinuclear heterochromatin and increase in cellular senescence – two conditions that correlate with dysregulation of TEs (Andrenacci et al., 2020). It can be assumed that in evolution, lamins arose as one of the protective mechanisms aimed at silencing TEs to protect hosts from genomic instability (Cavaliere et al., 2020). Therefore, laminopathies that cause premature aging and aging-associated pathology are most likely to cause activation of TEs as a key mechanism for the development of diseases (Andrenacci et al., 2020). Indeed, in an experiment on cell models expressing progerin characteristic of HGP, a pronounced increase in the expression of LINE, SINE, HERV and DNA transposons was revealed (Arancio, 2019).

## The relationship between lamins and transposons

Transposable elements are elements capable of transposition within the genome. There are two classes of TEs: retroelements (REs), transposed by reverse transcription and intermediate RNA; DNA-TEs, transposed using transposase by the mechanism of excision and insertion (Andrenacci et al., 2020). All TEs are subdivided into autonomous (contain genes of transposition enzymes) and non-autonomous (use protein products of other TEs for their transpositons). Retroelements are classified into those containing LTR (long terminal repeats) – LTR-REs and non-LTR-REs. The most common autonomous non-LTR-REs in the human genome are LINE1 (long interspersed nuclear elements), and nonautonomous ones are SINE (small interspersed nuclear elements). LTR-REs includes human endogenous retroviruses (HERV) (Arancio, 2019). Silencing of TEs is normally ensured by the degradation of their RNA and the formation of heterochromatin. Therefore, TEs are characterized by activation during aging of organisms as a result of ageassociated deregulation of heterochromatin and microRNA (Cavaliere et al., 2020).

The relationship between TEs activation and aging was found in termites. Reproductive queens live for tens of years without a significant increase in TEs expression, while working termites live for only a few weeks, which is due to TEs dysregulation in their genomes due to changes in piRNA expression (Elsner et al., 2018). Interestingly, in the somatic cells of Cnidaria, which are characterized by almost un-limited regeneration and immortality, piRNA and Piwi proteins are also expressed. It is accompanied by low levels of TEs activity in their genomes. These properties were revealed in Hydra vulgaris (Juliano et al., 2014). Piwi homologues named Cniwi are found at all stages of development of Podocoryne carnea not only in reproductive cells, but also in differentiated somatic cells (Seipel et al., 2004). Since the DNA repair systems in Cnidaria do not have a specific efficiency that distinguishes them from other animals, it can be assumed that the expression of piRNA in their organisms is the cause of delayed aging processes (Juliano et al., 2014).

During human aging, there is a progressive activation of LINE1, which leads to the activation of the interferon response due to the accumulation of LINE1 cDNA. These changes are the cause of aseptic inflammation and the activation of interferon observed during aging (De Cecco et al., 2019). At the same time, an increase in the level of proinflammatory cytokines in myeloid immune cells in the thymus during aging is accompanied by a gradual decrease in the level of lamin B1, which specifically functions for the correct organogenesis of the thymus by maintaining the expression of epithelial cell genes. It was found that a decrease in the level of lamin B1 leads to an increase in transcription of 533 genes and suppression of expression of 778 genes. Analysis of these genes showed their participation in cell adhesion, development of the immune system, differentiation of T-lymphocytes and cytokine production (Yue et al., 2019). Interestingly, chronic inflammation is also characteristic of PLs. In this case, the STAT1-regulated interferon-like response is induced by DNA hybrids: RNA through the signaling pathways cGAS (cyclic GMP-AMP synthase) and STING (stimulator of interferon genes) (Kreienkamp et al., 2018).

Transposable elements are the most important sensors of environmental stressors, exerting an adaptive regulatory effect on protein-coding genes (Mustafin, Khusnutdinova, 2019). According to the theory of “buffering function of the tail of lamins”, NL acts as an intracellular sensor of the reactive oxygen species (through conservative changes in the cysteine residue in the tail domain of the lamin). Throughout mammalian phylogeny, three cysteine residues (C522, C588, C591) at the C-terminus of lamin A are conserved. Of these, C588 and C591 are also characteristic of other vertebrates, and C522 is absent in non-mammalian animals.

Experiments on human skin fibroblasts showed that these amino acids in the functional tail domain of lamin A increase the sensitivity to reactive oxygen species, and their replacement with alanine promotes cellular aging (Pekovic et al., 2011). Thus, lamins and TEs are sensors for stressors that can cause their dysregulation. Moreover, abnormalities in the expression of TEs and lamins can potentiate each other, contributing to aging. Indeed, in experiments on Drosophila, the relationship between the age-related decrease in lamin B and the activation of various REs was proved (Chen H. et al., 2016).

Aging can be induced not only by decreased levels of lamins, but also by their increased production (Dreesen et al., 2013). REs are also characterized by similar properties. For example, in the study of 111 known REs in young and old Drosophila, not only an increase in the expression of 18 specific REs was determined, but also a decrease in the levels of other 18 REs (Chen H., et al., 2016). That is, with aging, there is not just an activation of TEs or a decrease in the levels of lamins, but a variety of changes in their expression, which potentiate each other.

The question arises as to what is primary – dysregulation of lamins or TEs. Investigation of the relationship between lamins and TEs could provide a basis for identifying the key mechanisms of physiological aging and for the development of new ways to prolong life. It is possible that changes in the levels of lamins and TEs expression during aging is a correlation rather than a causal relationship; however, the available data on their interconnections suggest a mutual potentiation of these structures during aging.

In different cells, LINE1s in the human genome are abundant in the lamin-associated domains of heterochromatin regions at the nuclear periphery. Sirtuin SIRT7 is involved in epigenetic transcriptional repression of LINE1 throughout the genome. The interaction of SIRT7 with A/C lamins plays an important role in this process. At the same time, SIRT7 provides deacetylation of H3K18, facilitating the interaction of LINE1s with NL. Deacetylation of H3K18 is insufficient to suppress LINE1s expression in the absence of lamins A/C, and depletion of lamins leads to transcriptional activation of LINE1s. That is, for the repression of LINE1s, their relationship with lamins A/C is necessary (Vazquez et al., 2019).

During the supercompactisation of differentiating human neutrophils genomes, centromeres, percentromeres, and LINEs move to the NL region. This property is retained for LINE in LBR-deficient cells, but is lost for other heterochromatin regions (Zhu et al., 2017). Human retroelements LTR7/HERV-H, LTR5_Hs, and L1HS form specific regulatory regions in the genome that selectively and site-specifically bind to lamin B1, as well as to NANOG homeobox, POU5F1, and CCCEC-binding factor (Glinsky, 2015).

There is also an inverse relationship where lamin B maintains the integrity of the nuclear genome by repressing TEs, which was found in experiments on Drosophila (Chen C.K. et al., 2016). Prelamin A is interconnected with TEs through BAF, which inhibits the integration of retroviruses. BAF is also found in protein complexes containing Sleeping Beauty transposase (Wang et al., 2014). BAF is required to modulate the effect of prelamin A on chromatin structure because it induces histone H3K9 trimethylation, as well as nuclear relocalization of the lamin-associated proteins LAP2-α and HP1-α (Loi et al., 2016).

## The relationship of lamins and transposable elements with viruses

Lamins (Pekovic et al., 2011) and transposons (Mustafin, Khusnutdinova, 2019) are sensors of environmental stress effects, characterized by their interconnection and possible potentiation of dysregulation during aging. Therefore, it is necessary to consider other possible mechanisms affecting their activity during aging. Environmental factors causing dysregulation of lamins and TEs include exogenous viruses. Determination of the role of lamins in viral infection can become the basis for identifying possible new ways of antiviral therapy, as well as the use of viral vectors for correcting aging processes by acting on specific lamins.

Induction of lamins A/C occurs in naive CD4+ T cells upon antigen recognition. In this case, lamins A/C act as a link between the nucleus and the plasma membrane during the activation of T cells. In experiments on mice, the role of lamins A/C in the response to vaccinia virus was revealed (Toribio-Fernandez et al., 2018). Proteins hnRNP and lamins A/C serve as carriers and mediators for the movement of p17 protein of avian reovirus between the nucleus and the cytoplasm (Chiu et al., 2019). The latency of HIV-1 after integration is characterized by reversible silencing of transcription driven by the LTR of the HIV genome. The lamin-interacting protein SUN2 maintains repressive chromatin and inhibits the transcription of proviral DNA, which is regulated by HIV LTR through its association with lamins A/C. Lamins A/C bind SUN2 to nucleosomes and to HIV-1 5′-LTR, which causes blockages of viral initiation and elongation of transcription (Sun et al., 2018).

The release of herpesviruses from the nucleus is accompanied by a change in the architecture of the NL (Vu et al., 2016). Herpes simplex virus-1 induces phosphorylation and reorganization of lamins A/C through the virulence factor – the product of the y134.5 gene. It allows the virus to exit through the nucleus (Wu et al., 2016). In turn, lamins facilitate the access of capsid to the inner nuclear membrane and the curvature of its sections around the capsid of the herpes virus during budding (Vu et al., 2016).

TRIM E3 ligase controls the replication of HSV-1 herpesviruses by affecting the integrity of lamins through changes in the structure of the host cell centrosomes. TRIM43 ubiquitinates the centrosome protein pericentrin, causing its proteasome degradation. It leads to lamins changes that suppress the active state of viral chromatin (Full et al., 2019). The mechanism of this phenomenon is due to the specific interaction of lamin A/C with the genomic DNA of the virus at the nuclear periphery. In this case, lamin inhibits the formation of heterochromatin in the region of HSV promoters (Silva et al., 2008).

Caspase-6-dependent dephosphorylation of lamins A/C is essential for SV40 virus penetration (Butin-Israeli et al., 2011). Baculovirus promotes phosphorylation of lamin B and destruction of NL during infection (Zhang et al., 2017). Canine parvovirus in the late stage of infection reorganizes NL with decreasing the levels of lamins A/C in the apical part of the nucleus (Mantyla et al., 2015).

The interrelation of lamins with viruses may indicate the possible participation of NL in the interconversion of viruses with TEs, as well as in transposon-controlled regulatory networks of genomes. Like lamins, TEs are involved in antiviral response, which may be related to their phylogenetic relationship. In the course of evolution, LTR-REs became sources of exogenous retroviruses (Xiong, Eickbush, 1990). ERV of various mammalian species are capable of converting into infectious form of viruses and converting back to REs (Zhuo, Feschotte, 2015). Expression products of LTRREs env genes cause restriction of exogenous retroviruses in animals (Malfavon-Borja, 2015). Retroelements enzymes can also be used to integrate exogenous viruses into host genomes (Speiseder et al., 2014). Exogenous viruses can regulate TEs activity. For example, human cytomegalovirus causes tissue-specific activation of LTR-RE (Assinger et al., 2013). MicroRNAs can serve as possible mediators in the interactions of viruses with TE and lamins. Investigation of microRNAs is promising both for the development of methods for slowing aging processes and for antiviral therapy

## Interrelation of lamins with transposons and miRNAs during aging

MicroRNA are involved in regulation of lamins (Sylvius et al., 2011; Toro et al., 2018) and TEs. It may be due to the origin of miRNAs in evolution from transposons. For the first time, evidence of the emergence of 55 different microRNAs from human TEs was obtained in 2007 (Piriyapongsa et al., 2007). In 2009, 73 human microRNAs derived from TEs were identified (Gu et al., 2009). In 2011, 191 microRNAs that arose from TE were identified (Filshtein et al., 2011). In the same year, another research group identified 226 human microRNAs derived from TE (MDTEs) (Yuan et al., 2011). In 2012, 235 human MDTEs were identified (Tempel et al., 2012), 409 MDTEs were identified in 2015 (Qin et al., 2015), 34 MDTEs were identified in 2020. In 2016, the MDTE database was created. This database contains information about microRNAs originated directly from TEs (Wei et al., 2016).

Aging is an important risk factor for the development of oncopathology. It may be due to common epigenetic mechanisms, since during aging, global DNA hypomethylation, TEs activation, and the development of genomic instability occur (Anwar et al., 2017). This assumption can be confirmed by data on the role of the same microRNAs in both carcinogenesis and aging. A lot of information has been accumulated on the association of various microRNAs with the development of tumors. Bioinformation database OncomiR was created (www.oncomir.org) (Wong et al., 2018). We analyzed 410 different microRNAs featured in OncomiR using the MDTE database (Wei et al., 2016). As a result, we found that 94 of these microRNAs were derived from TEs. It indicates the role of these microRNAs in the development of malignant tumors and the possibility of their use as a target for antitumor therapy.

Like TEs, lamins also play a role in carcinogenesis. It was shown that a deficiency of lamins A/C can be used as an independent risk factor for the development of cervical cancer (Capo-сhichi et al., 2016). Lamin B1 levels are significantly increased in patients with hepatocellular carcinoma (Abdelghany et al., 2018). Lamin B2 is highly expressed in non-small cell lung cancer and positively correlates with lymph node metastases. It is due to the interaction of lamin B2 with cyclin D1, which activates G9α expression and increases H3K9me2 levels. As a result, H3K9me2 binds to the promoter region of the E-сadherin CDH1 gene and stimulates cell migration (Zhang et al., 2020).

In gastric cancer, lamin B1 expression is reduced in tumor tissue, and low levels of lamin B1 are significantly correlated with clinical stage severity, depth of invasion, and poor prognosis. In the experiment, inhibition of lamin B1 causes the proliferation and migration of gastric cancer cells, which is due to the activation of the PI3K/PTEN/Akt and MAPK/ERK pathways, as well as the suppression of p53/ h21WAF1/CIP1 (Yu et al., 2020).

Low levels of lamins A/C expression in cancer are specific for poor prognosis. In breast cancer (BC), the loss or decrease in the expression of lamins A/C was significantly associated with large tumor sizes, poor prognosis and the development of long-term results (reduced survival time) (Alhudiri et al., 2019). Comparison of osteosarcoma and osteoblast cell lines showed a lower level of lamin A expression in osteosarcoma cells. Elevated lamin A levels reduce cancer cells’ ability to migrate (Evagelisti et al., 2020). Thus, carcinogenesis is associated both with an increase and (most often) with a decrease in lamin expression, that is, it is explained by their dysregulation.

This is due to the global regulatory role of lamins in the expression of various genes. Therefore, it is important to determine the specific features of changes in the expression of lamins in each type of tumor in order to target them during antitumor therapy. Most tumors are characterized by decreased lamins expression, which may be one of the key mechanisms of initiation and maintenance of carcinogenesis, because lamins serve as host defense systems for TEs silencing. Therefore, the loss of control by the lamins leads to pathological activation of TEs with subsequent genomic instability and tumor development. The effect of increased levels of lamins on tumor development is probably due to the possibility of opposite regulatory effects of lamins on TEs, depending on localization of TEs in active or repressed genome regions (Cavaliere et al., 2020).

Like transposons, during aging and carcinogenesis, lamins are also associated with changes in microRNA levels. For example, the target of the oncogenic miR-129, the expression of which is increased in BC, is lamin A. The target of the oncogenic miR-218, the level of which decreases in BC, is lamin B1 (Setijono et al., 2018). LMNA expression in breast cancer cells is suppressed by miR-9, which leads to the progression of the disease due to changes in the deformability of the nucleus and an increase in the invasive ability of cells (Guinde et al., 2018). MiR-122 inhibits hepatocellular cancer cell proliferation by suppressing the expression of lamin B2 (Li et al., 2019). MiR-351-5p regulates lamin B1 expression and promotes phloxuridine-induced tumor cell apoptosis (Sato et al., 2020). In fibroblasts associated with BC, the expression of miR-222 is increased (compared to normal cells). The direct target of this microRNA is LBR. Therefore, miR-222 is considered for BC targeted therapy (Chatterje et al., 2019).

During human aging, the levels of LMNB1 protein products in fibroblasts and keratinocytes of the skin decrease, which is mediated by the influence of miR-23a (Dreesen et al., 2013). An increase in LMNB1 expression due to gene duplication in autosomal dominant leukodystrophy in adults leads to progressive brain demyelination. This is due to the role of lamin B1 in regulation of myelin formation and maintenance during aging. The targets of miR-23a are also the PTEN gene transcript (phosphatase and tensin homolog on chromosome 10) and long noncoding RNA 2700046G09Rik (Lin et al., 2014). MiR-124-3p complementarily binds to 3′-UTR of LMNA gene, suppressing its expression (Bao et al., 2019). Activation of miR-141-3p during replicative senescence decreases the activity of HDAC1 and HDAC2. MiR-141-3p has a targeted regulatory effect on ZMPSTE24 endoprotease prelamin A (Yu et al., 2013).

Like TEs, miRNAs affect lamins according to the principle of mutual regulation, since miRNAs genes are located in genome regions associated with lamins. For example, in mice, aging-associated miRNA genes are located on the X chromosome in a cluster located in the lamin-associated domain (Elias et al., 2019). Long noncoding RNA Xist is involved in X chromosome inactivation by directly interacting with the LBR and recruiting it to the NL. It leads to remodeling of the three-dimensional structure of DNA, which allows Xist to distribute along the X chromosome and cause silencing of transcriptionally active genes (Chen C.K. et al., 2016).

In skeletal muscles of patients with muscular dystrophies caused by mutations in LMNA gene, pronounced dysregulation of sixteen different microRNAs was revealed, which are involved in Wnt-signaling pathways, MAPK, and the regulation of transforming growth factor β. Nine of these microRNAs are involved in regenerative processes (miR-100, -127-3p, -148a, -136 *, -192, -335, -376c, -489, -502-3p) and are detected at high levels in fetal muscles (Sylvius et al., 2011). Expression of the mutant allele of LMNA gene (mutation R482W) is accompanied by an increase in levels of miR-335, which has antilipogenic properties (Briand et al., 2018). Significantly increased expression of let-7a-5p, miR-142-3p, miR-145-5p, and miR-454-3p is observed in patients with familial dilated cardiomyopathy associated with mutations in LMNA (Toro et al., 2018).

According to the MDTE database, among the miRNAs associated with lamina pathology, the origin from LINE2 was proven for miR-192 and miR-502, the origin from MIR (SINE-RE) was proven for miR-335 (Wei et al., 2016). It should be noted that miR-335 also plays a role in human physiological aging and age-associated neurological pathology (Raihan et al., 2018). It may be due to the role of lamins in protecting genomes from transpositions (Cavaliere et al., 2020), since TEs are involved in the epigenetic regulation of lamins. That is, the structural and functional relationship between lamins and TEs is mutually regulatory. On the one hand, lamins are involved in the control of TEs activity. On the other hand, TEs can exert an epigenetic regulatory effect on NL, which may be due to the influence of microRNAs derived from TEs.

## Conclusion

Lamins play an important role in driving gene expression and in TEs silencing, thereby preventing genome instability. Therefore, dysregulation of lamins, mainly associated with inactivating mutations in their genes or a decrease in their expression, leads to TEs activation. Therefore, pathological activation of TEs and the resulting genomic instability play an important role in changing the levels of lamins in malignant neoplasms. The role of lamins in viral infection supports this assumption, since TEs are phylogenetically associated with viruses.

Transposable elements and lamins share common properties, such as dysregulation during aging and carcinogenesis, as well as interactions with microRNAs. Mutual regulation of lamins and TEs testifies to the mutual potentiation of their dysregulation in laminopathies, carcinogenesis and physiological aging. Identification of key changes in TEs and miRNAs derived from them can become the basis for targeted therapy of laminopathies, malignant neoplasms and for prolonging life. Investigation of the role of lamins in the interaction with TEs and viruses is also a promising direction in the development of antiviral therapy and vaccination.

## Conflict of interest

The authors declare no conflict of interest.
